# Restoring the encoding properties of a stochastic neuron model by an exogenous noise

**DOI:** 10.3389/fncom.2015.00042

**Published:** 2015-05-06

**Authors:** Alessandra Paffi, Francesca Camera, Francesca Apollonio, Guglielmo d'Inzeo, Micaela Liberti

**Affiliations:** ^1^Department of Information Engineering, Electronics and Telecommunications, Sapienza University of RomeRome, Italy; ^2^Italian Inter-University Center for the Study of Electromagnetic Fields and Biological SystemsGenova, Italy

**Keywords:** single neuron, HH model, electric stimulation, exogenous noise, stochastic resonance, signal detection

## Abstract

Here we evaluate the possibility of improving the encoding properties of an impaired neuronal system by superimposing an exogenous noise to an external electric stimulation signal. The approach is based on the use of mathematical neuron models consisting of stochastic HH-like circuit, where the impairment of the endogenous presynaptic inputs is described as a subthreshold injected current and the exogenous stimulation signal is a sinusoidal voltage perturbation across the membrane. Our results indicate that a correlated Gaussian noise, added to the sinusoidal signal can significantly increase the encoding properties of the impaired system, through the Stochastic Resonance (SR) phenomenon. These results suggest that an exogenous noise, suitably tailored, could improve the efficacy of those stimulation techniques used in neuronal systems, where the presynaptic sensory neurons are impaired and have to be artificially bypassed.

## Introduction

Different techniques for the stimulation of neuronal systems have been developed. Some are based on magnetic coupling, such as the Transcranial Magnetic Stimulation (TMS) (Corthout et al., 2001) and low-intensity magnetic stimulation (Di Lazzaro et al., 2013), while others use electric fields, such as Deep Brain Stimulation (DBS) (Okun et al., 2012; Paffi et al., 2013a), Functional Electric Stimulation (FES) of peripheral nerves (Peckham and Knutson, 2005), cochlear prostheses (Wilson et al., 1991; Clark, 2003), and Intracortical Microstimulation (ICMS) (Brock et al., 2013; Overstreet et al., 2013).

Despite great interest in such applications and the experimental activities to evaluate the effect of electromagnetic fields on single neurons and networks (Marchionni et al., 2006; Platano et al., 2007; Ahmed and Wieraszko, 2009; Moretti et al., 2013), the mechanisms of action are not clearly understood (Apollonio et al., 2013; Di Lazzaro et al., 2013) and the techniques are not yet optimized.

Theoretical studies to understand neuronal system functioning are based on biophysical models. At the single neuron level, a lot of work has been done using simple (Mainen et al., 1995; Rapp et al., 1996; Rinzel and Ermentrout, 1998) or augmented (Tateno et al., 1998; Pospischil et al., 2008) Hodgkin and Huxley (HH) descriptions (Hodgkin and Huxley, 1952), both under physiologic conditions and the action of exogenous stimulations (Mino et al., 2004; Giannì et al., 2005, 2006; Camera et al., 2012, 2013).

The HH model is a nonlinear active circuit, which behaves as an oscillator if the injected constant current (stimulation current) overcomes a threshold (Rinzel and Ermentrout, 1998). Such a current represents a physiologic stimulation from all the presynaptic neurons. Depending on this parameter, the HH model can display a stable resting state or/and a stable limit cycle (Hassard, 1978), corresponding to a periodic oscillation of the membrane voltage in the form of a spike train.

However, the HH model does not adequately take into account the stochastic behavior of neurons. Electrophysiology recordings have shown that actual neurons have an intrinsic stochastic behavior (Sigworth, 1980; Mainen and Sejnowski, 1995; Dorval and White, 2005), shown by the unreliable responses and non-deterministic current thresholds for firing. This is due to the noisy environment of the neuron, in particular to the intrinsic stochasticity of channel gating (channel noise) (White et al., 2000). The level of this noise decreases with the number of ionic channels and so depends on the channel density and the size of the neuron considered (Schneidman et al., 1998; White et al., 2000).

Several authors have provided stochastic neuron models (Schneidman et al., 1998; White et al., 2000) to study how channel noise may improve the encoding of a physiologic or artificial input stimulation (Schneidman et al., 1998; Jung and Shuai, 2001; Manwani et al., 2002; Mino et al., 2004; Giannì et al., 2005, 2006, 2007; Woo et al., 2010) with different frequency content (Bulsara et al., 1993; Longtin, 1993; Liu et al., 1999; Rudolph and Destexhe, 2001; Yu et al., 2001a; Giannì et al., 2005, 2006, 2007; Liberti et al., 2009a; Sengupta et al., 2013). This positive role of the endogenous noise in signal encoding and processing observed in neuronal models was often attributed to the Stochastic Resonance (SR) phenomenon (Gammaitoni, 1998; Moss et al., 2004; McDonnell and Abbott, 2009).

However the endogenous noise is essentially related to the type of neuron and is difficult to manipulate and control. From a biomedical perspective it is more interesting to use theoretical models to elucidate the role of an artificial noise externally applied.

In previous studies, we showed that a suitably tailored exogenous noise could increase firing activity and improve signal detection through the SR mechanism in compartmental models of neuronal systems with reduced levels of endogenous noise (Paffi et al., 2006, 2007, 2013b). Here we extend our idea to systems where the presynaptic stimulation was lowered due to impairment of sensory neurons that have to be artificially bypassed at different levels of the neuronal pathway toward the cortical region of sensorial processing. Examples are the cochlear prostheses, where an electrode inserted in the cochlea directly stimulates the fibers of the auditory nerve (Wilson et al., 1991; Clark, 2003), and ICMS to deliver sensory perceptions to the auditory or visual cortex (Brock et al., 2013; Overstreet et al., 2013).

To test our idea for the optimization of stimulation techniques, a simple and well-characterized HH neuron model is considered. The normal functioning is modeled with a suprathreshold input current, and pathologic conditions with a subthreshold presynaptic stimulation. Different kinds of neurons of the sensory pathway, characterized by different sizes and, hence, by different levels of endogenous noise, are accounted for by changing the number of ionic channels.

The first step is to demonstrate that the detectability of the exogenous stimulation signal is degraded in impaired sensory neurons as a function of the endogenous noise level. Then we want to show that a suitably tailored exogenous noise can partially restore the signal encoding in the impaired neurons, in agreement with the SR phenomenon.

The main aim of this paper is to show that the reduced encoding capability of pathologically understimulated neuronal systems can be improved using an exogenous noise, opening the way for prosthetic applicators delivering the exogenous stimulation signal and noise.

The paper is organized as follows. In Section Materials and Methods the stochastic neuron model is described (Section Stochastic Neuron Model), together with the methods for introducing the exogenous electric signal (Section Introduction of the Exogenous Signal) and noise (Section Introduction of the Exogenous Noise) and for evaluating neuron excitability and signal encoding (Section Observables). Results are presented and discussed in Section Results, without (Section Encoding Features of the Model) and with the exogenous noise (Section Role of the Exogenous Noise). Finally, in Section Discussion and Conclusions, results are discussed and conclusions are given.

## Materials and methods

### Stochastic neuron model

We use a stochastic neuron model (Fitzhugh, 1965; Sigworth, 1980; Clay and DeFelice, 1983; Schneidman et al., 1998), derived from the HH model (Hodgkin and Huxley, 1952).

**Figure 1:**
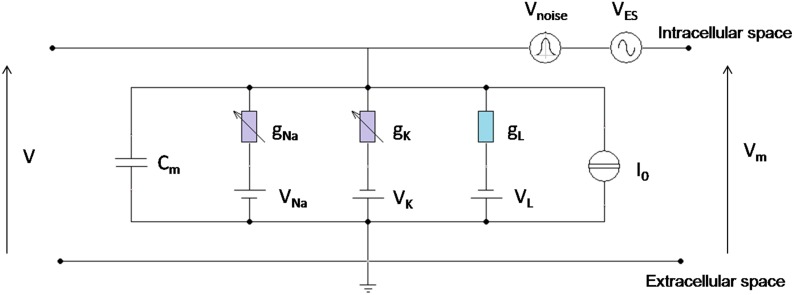
**Circuital representation of the neuron model**. C_m_ is the specific membrane capacitance; g_L_ is the leakage specific conductance; g_Na_ and g_k_ are Sodium and Potassium specific conductances, voltage (V_m_) dependent; I_0_ is the specific stimulation current; V_Na_, V_K_, V_L_ are the reversal potentials for Sodium, Potassium, and leakage currents, respectively; V_ES_ and V_noise_ are the voltage perturbations, due to the exogenous electric signal and the exogenous Gaussian noise, superimposed to the physiological membrane voltage V; V_m_ is the total voltage between the intracellular and the extracellular space.

The equivalent scheme is shown by the parallel combination of five current branches, as in Figure 1.

Sodium and Potassium currents were calculated using a channel-state-tracking algorithm (Mino et al., 2002) where the ionic channels are modeled as the combination of independent gating particles whose dynamics is well-described by Markov chains (Rubinstein, 1995; Mino et al., 2002).

The number of voltage-gated channels belonging to a given population determines the level of endogenous noise in the system (Schneidman et al., 1998; White et al., 2000). This decreases with the square root of the channel number and, for a given channel density, with the size of the neuron considered (Schneidman et al., 1998; White et al., 2000).

Therefore, the endogenous level of noise is typical of each kind of neuron and strongly varies with neuron size.

For example, the auditory fibers have a small diameter estimated between 1 and 9 μm in (Engstrom and Wersall, 1958), or between 1 and 5 μm in Gleich and Wilson (1993). The cell body is larger, with a cross-sectional area of 300 μm^2^ (Liberman and Oliver, 1984; Woo et al., 2010). At the brainstem in the cochlear nucleus, the bushy cells that receive inputs from the auditory fibers range between 300 and 1200 μm^2^ in area (Sento and Ryugo, 1989). Cortical neurons of Layer I have the soma size ranging from 7 to 17 μm in diameter (Hestrin and Armstrong, 1996), corresponding to an area from 154 to 900 μm^2^, if one assumes a spherical morphology.

In this work, membrane patches of 200, 300, and 600 μm^2^ have been considered, corresponding to N_Na_ = 12,000, N_Na_ = 18,000, and N_Na_ = 36,000, respectively, for constant channel densities of 18 N_K_/μm^2^ and 60 N_Na_/μm^2^ (Hodgkin and Huxley, 1952).

A neuron of 200 μm^2^ could represent the largest axon in the auditory fiber (Engstrom and Wersall, 1958) or a small-sized neuron of Layer I in the cortex (Hestrin and Armstrong, 1996); one at 300 μm^2^ could be the smallest bushy cells in the cochlear nucleus (Sento and Ryugo, 1989) or a medium-sized cortical neuron of Layer I, and neurons of 600 μm^2^ represent a typical area of bushy cells and cortical neurons.

The current generator I_0_ in Figure 1 was set to different values: I_0_ = 2 μA/cm^2^, I_0_ = 4 μA/cm^2^, I_0_ = 7 μA/cm^2^, below or above the firing threshold (I_0th_ = 6.3 μA/cm^2^) of the associated deterministic oscillator (Rinzel and Ermentrout, 1998).

Considering I_0_ as the total stimulation from the presynaptic neurons (presynaptic current), particularly from the sensory receptors, these conditions can be representative of a neuron normally stimulated by the sensory inputs (I_0_ = 7 μA/cm^2^) or a neuron where the receptors stimuli are slightly (I_0_ = 4 μA/cm^2^) or significantly (I_0_ = 2 μA/cm^2^) reduced. This is a typical impairment induced by aging, direct damage, or degenerative diseases of the sensory receptors such as the cells of the organ of Corti in the Cochlea (Ritter et al., 1981).

The neuron stochastic model used HH parameters (Hodgkin and Huxley, 1952) in the C++ environment using the Forward Euler integration method with time steps of 10 μs.

The output of the model is the time course of the voltage across the membrane V(t).

### Introduction of the exogenous signal

There is consensus in literature that the effect of an exogenous magnetic or electric stimulation delivered by a coil or an implanted electrode is the creation of an electric field in the tissue that in turn induces a perturbation on the neuron membrane voltage (Foster and Schwan, 1986; Mino et al., 2004; Giannì et al., 2006; Merla et al., 2012). Therefore, unless a current is directly injected across the membrane, the interaction between the exogenous signal and the neuron membrane must be inserted as a voltage generator in series with the neuron circuital model (Figure 1), as already done in number of studies (Tsong and Astumian, 1987; Mino et al., 2004; Giannì et al., 2006; Woo et al., 2010; Paffi et al., 2013b). As previously discussed (Giannì et al., 2006), this additive voltage can describe the non-linear interaction between the exogenous signal and the neuron activity (Stodilka et al., 2011), since it induces a perturbation in the dynamics of the voltage-dependent Sodium and Potassium channels. The effects on the ionic currents are temporally integrated and reflected back on the membrane potential, showing a feedback interaction mechanism (Apollonio et al., 2000).

A weak deterministic sinusoidal signal, of amplitude V_ES_ = 500 μV was considered for frequencies between 10 and 500 Hz. The term “weak” means that it does not induce firing activity in the subthreshold neuron, provided no sources of stochasticity, endogenous (channel noise), or exogenous, are present.

### Introduction of the exogenous noise

The endogenous noise, due to the neuron size, i.e., the number of ionic channels, is an intrinsic feature of each neuron type and cannot be artificially tuned or modulated. Conversely, a well-defined exogenous noise can be added to the sinusoidal signal and suitably tuned in terms of power and frequency content.

In the circuit scheme of Figure 1 the exogenous noise was modeled as a random voltage source whose level is given by, $V_{noise}=\sqrt{2D}\times \zeta \left(t\right)$ where ζ(*t*) is a Gaussian process with zero mean and unitary variance. Accordingly, the noise power D is measured in mV^2^ and was varied in the range [0.7-25] (mV^2^).

The well-known equation describing the current balance of the HH circuit in the presence of an exogenous signal and noise shown in Figure 1, becomes:

$$\begin{array}{l} C_{m}\left(\frac{dV_{m}}{dt}+\frac{dV_{ES}}{dt}+\frac{dV_{noise}}{dt}\right)=-g_{Na}(V_{m}+V_{ES}\\ +V_{noise}-V_{Na})-g_{K}\left(V_{m}+V_{ES}+V_{noise}-V_{K}\right)-g_{L}(V_{m}\\ +V_{ES}+V_{noise}-V_{L})+I_{0} \end{array}$$

As shown by Paffi et al. (2013b) the voltage noise was filtered to obtain a Lorentzian Power Spectral Density (PSD):

$$PSD\left(f\right)=\frac{A}{{\left(\omega_{c}\right)}^{2}+{\left(2\pi f\right)}^{2}}$$

The use of a Lorentzian behavior is a straightforward choice since, under the passive linear approximation (Steinmetz et al., 2000), the neuronal membrane behaves like a single pole filter with a time constant equal to the membrane capacitance divided by the total conductivity of the ionic channels in the patch (Rinzel and Ermentrout, 1998).

The cutoff angular frequency ω_c_ = 2.5 × 10^3^ rad/s of the Lorentzian filter was chosen on the basis of theoretical calculations (Paffi et al., 2013b) and numerical simulations (Paffi et al., 2007).

**Figure 2:**
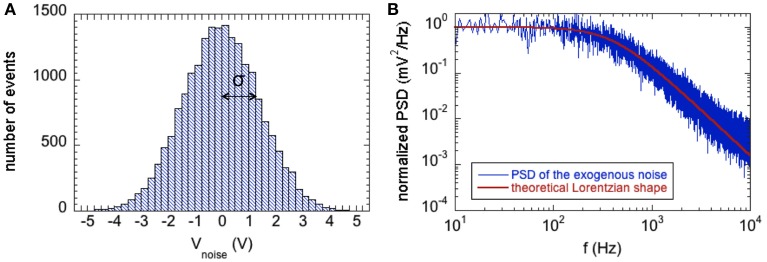
**(A)** Histogram showing the zero-mean Gaussian distribution of the exogenous voltage noise with power *D* = 2 mV^2^; the standard deviation (σ) of the distribution is equal to the square root of D. **(B)** Normalized Power Spectral Density of the exogenous voltage noise (blue line) compared with the theoretical Lorentzian spectrum with cutoff angular frequency ω_c_ = 2.5 ×10^3^ rad/s (red line).

Figure 2 shows the features of the exogenous voltage noise in terms of the Gaussian distribution (Figure 2A) and correlation properties by the PSD (Figure 2B). The PSD was estimated as the Periodogram averaged over 10 runs of the Gaussian process 1 s long.

Such noise can increase the firing activity of the subthreshold neurons as already demonstrated by Paffi et al. (2013b).

### Observables

Due to the stochasticity of the model, the neuron properties, such as the number of spike per second or the frequency content of the spike sequence, can be calculated only as statistical values over a population of R runs of the model.

To quantify the excitability of the neuron, the average number of spikes per second was calculated over 100 V(t) traces 1 s long, together with the standard error.

To determine the “time encoding” of the neuron model, that is, the capability of encoding different input signals in the spike timing within the firing sequence, the Periodogram has been used as a spectral estimator. As a preliminary step to retain information only on the sequence of spikes (Giannì et al., 2005, 2006), disregarding their shape, the time course of the membrane voltage V(t) over 1 s, has been converted into a time series of Dirac pulses U(t), each corresponding to a spike, having height 100 mV as suggested in Gluckman et al. (1996); Levin and Miller (1996) and Yu et al. (2001a,b).

The PSD, averaged over *R* runs (*R* = 100 in the presence of the channel noise alone; *R* = 300 with the presence of channel and exogenous noise) of the signal U(t), has been calculated using the Fast Fourier Transform (FFT) algorithm. It is worth noting that in the presence of both noises the spectral estimator with *R* = 100 was not satisfactory due to high variance; so to calculate results of Section Role of the Exogenous Noise it was necessary to increase the number of runs from 100 to 300. For each frequency point, the standard error of the PSD was associated with the average value.

**Figure 3:**
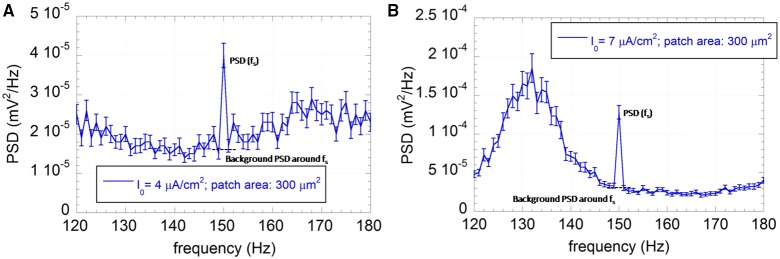
**Average PSD with the associated standard error (*R* = 100) of the output sequence U(t) for the neuron model with patch area 300 μm^2^ and I_0_ = 4 μA/cm^2^ (A) or I_0_ = 7 μA/cm^2^ (B)**. The applied exogenous signal is a sinusoid at 150 Hz, 500 μV of amplitude.

The signal to noise ratio (SNR), calculated as the ratio between the strength of the peak of the average spectrum at the forcing frequency (f_s_) and the background average spectrum around the same frequency (Figure 3) (Gluckman et al., 1996; Levin and Miller, 1996; Gammaitoni, 1998; Yu et al., 2001a,b; Giannì et al., 2006; Paffi et al., 2013b), was used to evaluate the signal detectability as a function of the signal frequency or the exogenous noise level. The background spectrum at the signal frequency was estimated as the average between the values assumed by the PSD 1 Hz before and 1 Hz after the signal frequency (Gammaitoni, 1998; Giannì et al., 2006; Paffi et al., 2013b). The standard error of the SNR was calculated using the standard errors propagation of the correlated variables PSD(f_s_), PSD(f_s_–1), PSD(f_s_ + 1). The correlation coefficient of these adjacent samples was calculated to be around 0.9.

Matlab functions have been used to extract the aforementioned observables from the model output.

## Results

### Encoding features of the model

It is known from literature that HH models exhibit a frequency sensitivity that depends on the model parameters, particularly on the constant input current I_0_ (Liu et al., 1999; Yu et al., 2001a; Giannì et al., 2006).

Here we examine and compare the encoding capability of the neuron model when I_0_ assumes values of 2, 4, and 7 μA/cm^2^ and the patch area, determining the endogenous noise, is equal to 200, 300, or 600 μm^2^.

As described in Section Stochastic Neuron Model, the exogenous signal was a deterministic sinusoid of amplitude 500 μV and frequency spanning from 10 to 500 Hz, applied as a voltage perturbation over the neuron membrane.

**Figure 4:**
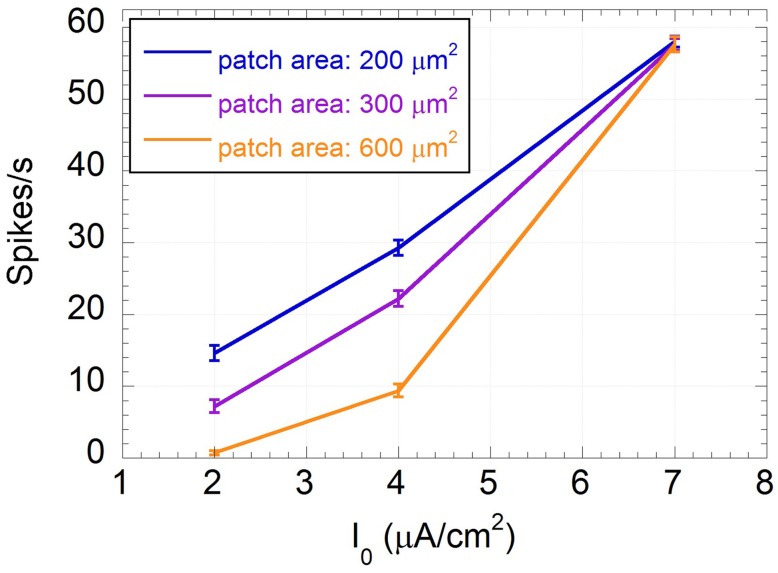
**Mean number of spikes per second and standard error calculated over the frequency of the input sinusoidal signal vs. the input constant current I_0_ for patch areas: 200 (blue line), 300 (purple line), and 600 (orange line) μm^2^**.

As already shown (Paffi et al., 2013b), the weak exogenous signal does not affect the neuron excitability, i.e., the number of spikes per second, independently of the frequency considered. However, this value significantly changes with the constant input current I_0_ and the endogenous noise level, as shown in Figure 4, where the number of spikes per second, averaged over the results obtained for all frequency values, are displayed together with the standard errors.

From Figure 4 it is evident that the standard errors on the number of spikes are very small with respect to variations due to I_0_ or to the patch areas, confirming that, when the applied signal is as low as 500 μV, the frequency encoding mechanism is not likely to occur.

As expected, the “impaired” neurons (I_0_ = 2 μA/cm^2^, I_0_ = 4 μA/cm^2^) are much less stimulated than the “healthy” one (I_0_ = 7 μA/cm^2^). This reduction in firing activity is likely to negatively affect the neuron encoding capability. In particular, the “severely impaired” neuron for the biggest patch area (600 μm^2^) is almost silent, losing any possibility of signal detection. For the “healthy” neuron the endogenous noise does not change the excitability that is completely determined by the input current; conversely, in subthreshold conditions, i.e., for the “impaired” neurons, the higher the endogenous noise level the higher the firing rate.

This shows that different neurons may be more or less sensitive to impairment depending on their channel noise. That noise has a beneficial effect on the firing activity of “impaired” neurons, suggesting a positive role of an exogenous noise.

If one considers the total power of the sequence U(t) 1 s long (Table 1), the same behavior is observed. The output power does not depend on the patch area for I_0_ = 7 μA/cm^2^ but increases up to 17 times for I_0_ = 2 μA/cm^2^ if the patch area decreases from 600 to 200 μm^2^.

**Table 1:** **Total power (mV^2^) of the output sequence U(t) 1 s long calculated for the different values of patch areas and input currents**.

**Patch area (μm^2^)**	**I_0_ = 2 μA/cm^2^**	**I_0_ = 4 μA/cm^2^**	**I_0_ = 7 μA/cm^2^**
200	0.68	1.42	2.85
300	0.32	1.06	2.83
600	0.04	0.42	2.83

Time encoding performances of the neuron model were measured using the SNR, as described in Section Observables. Indeed, if time encoding occurs, the PSD of U(t) will present a component at the signal frequency higher than the background level around the signal frequency, as in Figure 3 for a 150 Hz signal, a patch of 300 μm^2^, input currents I_0_ = 4 μA/cm^2^ (Figure 3A) and I_0_ = 7 μA/cm^2^ (Figure 3B).

Figure 3 shows that the exogenous signal can synchronize some spike events with its own frequency, leading to a frequency peak at 150 Hz emerging from the background PSD with a consequent SNR value greater than one.

**Figure 5:**
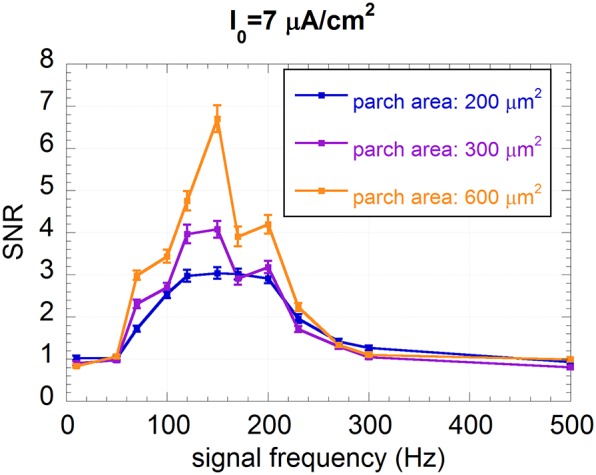
**Mean SNR and standard error (*R* = 100) vs. the signal frequency for I_0_ = 7 μA/cm^2^ and membrane patches of 200, 300, and 600 μm^2^; the signal is a sinusoid with amplitude V_ES_ = 500 μV and frequency ranging from 10 to 500 Hz**.

The frequency sensitivity of the “healthy” neuron, from 10 to 500 Hz, is plotted in Figure 5 for the three patch areas. At first one can observe a bell-shaped behavior of the SNR, with a peak centered around 150 Hz. As the patch area decreases, i.e., the endogenous noise increases, the curves become smoothed and the maximum SNR decreases. This indicates that endogenous noise has a detrimental effect on the encoding capability in the healthy neuron.

**Figure 6:**
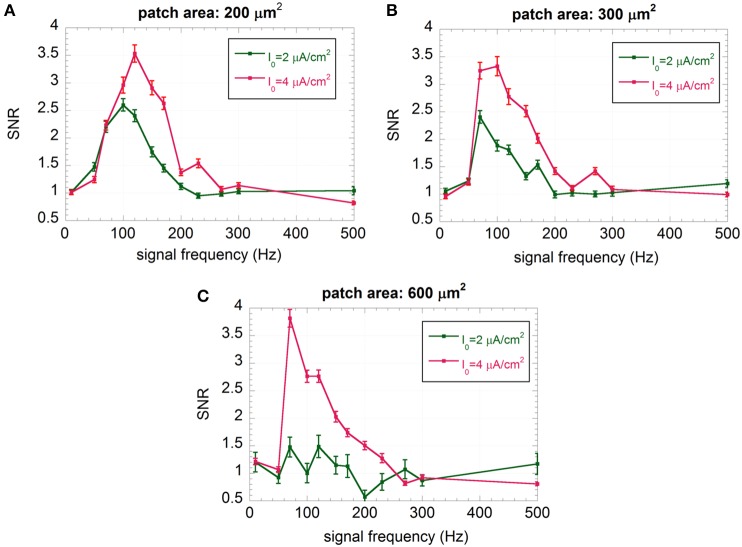
**Mean SNR and standard error (*R* = 100) vs. the signal frequency for I_0_ = 2 and 4 μA/cm^2^ and membrane patches of 200 μm^2^ (A), 300 μm^2^ (B), and 600 μm^2^ (C); the signal is a sinusoid with amplitude V_ES_ = 500 μV and frequency ranging from 10 to 500 Hz**.

Looking at the “impaired” neurons (Figure 6), the maximum of the curve shifts toward lower frequencies and the SNR at 150 Hz, where the “healthy” neuron exhibits the maximum sensitivity, significantly decreases (always below 3). For the “severely impaired” neuron and the largest size, the signal becomes undetectable (SNR almost equal to one with higher error bars), since the firing activity is almost completely suppressed. From a biophysical point of view, this can be interpreted as a severe worsening in neuron performances if the afferent inputs are lacking. Only the smallest neuron, for I_0_ = 4 μA/cm^2^ shows an encoding capability at 150 Hz similar to that of the “healthy” neuron with the same size, suggesting again a positive role of the noise in understimulated neurons.

With an exogenous sinusoidal signal of 150 Hz and amplitude 500 μV, in Section Role of the Exogenous Noise we investigated the possibility of restoring degraded performances by adding a correlated noise to the system from the outside.

### Role of the exogenous noise

As shown in Section Encoding Features of the Model, the effect of the impairment of the afferent stimulation is a drastic reduction of the neuron firing and signal encoding, especially for neurons with a lower endogenous noise, i.e., the larger ones.

From a biomedical perspective the question arises as to whether the exogenous noise can improve the firing activity and the encoding capability of the “impaired” neurons in terms of number of spikes per second and SNR. Therefore, we have added the correlated voltage noise described in Section Introduction of the Exogenous Noise to the neuron models in subthreshold conditions (I_0_ = 2 and 4 μA/cm^2^).

Among the six different conditions shown in Figure 6, we have not considered the patch of 600 μm^2^ with I_0_ = 2 μA/cm^2^, since the neuron has no residual activity, and the patch of 200 μm^2^ with I_0_ = 4 μA/cm^2^, since the encoding performances at 150 Hz are already comparable to those of “healthy” neurons with the same size.

**Figure 7:**
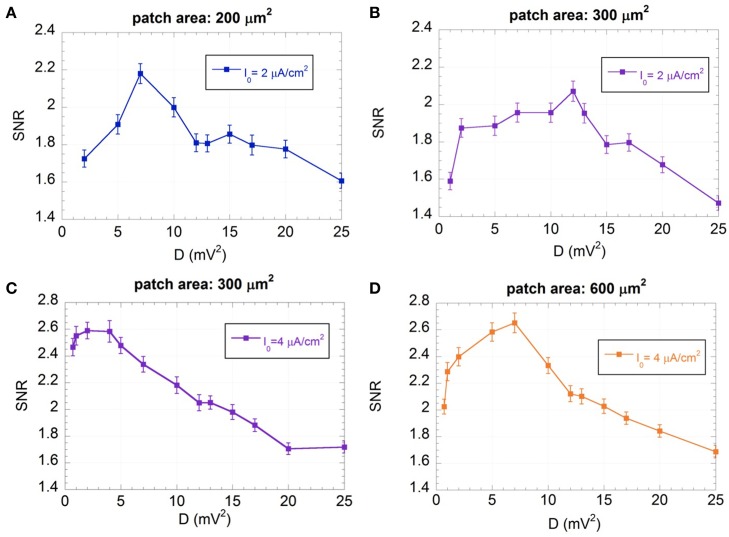
**Mean SNR and standard error (*R* = 300) as a function of the variance of the exogenous noise (D) for I_0_ = 2 μA/cm^2^ and membrane patches of 200 (A) and 300 μm^2^ (B), and for I_0_ = 4 μA/cm^2^ and membrane patches of 300 μm^2^ (C) and 600 μm^2^ (D); the signal is a sinusoid with amplitude V_ES_ = 500 μV and frequency f = 150 Hz; the exogenous noise is a zero mean Gaussian process with a Lorentzian spectrum (ω_c_ = 2.5×10^3^ rad/s)**.

The SNRs as a function of D are shown in Figure 7 for the “severely impaired” neuron (I_0_ = 2 μA/cm^2^) with area 200 (Figure 7A) and 300 μm^2^ (Figure 7B) and for the “impaired” neuron (I_0_ = 4 μA/cm^2^) with area 300 μm^2^ (Figure 7C) and 600 μm^2^ (Figure 7D).

Figure 7 shows that, for all the conditions considered, there exist noise levels that improve the SNR, showing the typical behavior of SR (Gammaitoni, 1998; Moss et al., 2004; McDonnell and Abbott, 2009). For each condition it is possible to identify an optimum noise level (D_opt_), where the SNR is maximum, that depends on the membrane patch and the input current I_0_. In particular, for the same I_0_, D_opt_ is lower for the smaller patch area, showing that the higher the channel noise, the lower the exogenous noise to be supplied, in agreement with previous results (Schmid et al., 2001; Paffi et al., 2013b).

Table 2 summarizes, for each condition studied, the SNR values exhibited by the model at 150 Hz without the endogenous noise and with the optimum noise power (D_opt_) reported in the last column. The table shows that the SNR increases in the presence of the optimum exogenous noise. This improvement is not significant for the neuron with I_0_ = 4 μA/cm^2^ and membrane patch 300 μm^2^, where the encoding performances without the exogenous noise (SNR = 2.51 ± 0.10) are still acceptable due to high levels of endogenous activity. In all the other cases, one may observe a significant increase in SNR by up to 57%.

**Table 2:** **SNR values exhibited by the neuron model at 150 Hz without and with the exogenous noise at the optimum level D_opt_**.

**Patch area (μm^2^)**	**Input current (μA/cm^2^)**	**SNR**		**D_opt_ (mV^2^)**
		**Without exogenous noise**	**With optimum exogenous noise**	
200	2	1.74±0.09	2.18±0.05 (25%)	7
300	2	1.32±0.06	2.07±0.05 (57%)	12
300	4	2.51±0.10	2.59±0.06 (3%)	2
600	4	2.02±0.12	2.66±0.07 (32%)	7

Although the performances of the “impaired” neuron are not been completely restored, they are significantly improved, confirming the potentially beneficial effect of an exogenous noise according to the SR paradigm.

**Figure 8:**
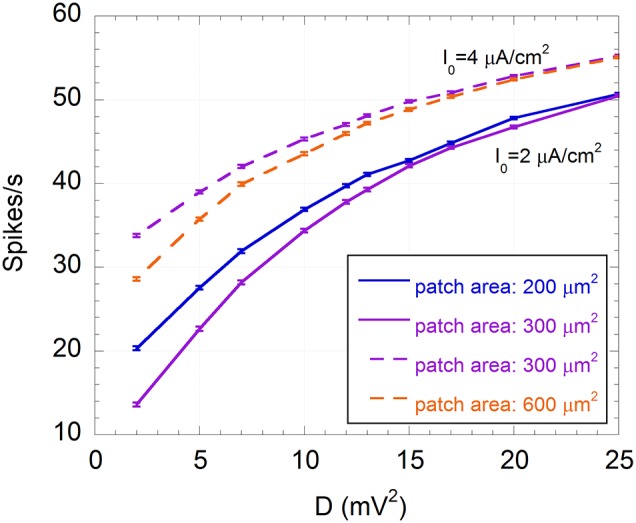
**Mean number of spikes per second and standard error (*R* = 300) as a function of the variance of the exogenous noise (D) for I_0_ = 2 μA/cm^2^ and membrane patches of 200 μm^2^ (solid blue line) and 300 μm^2^ (solid purple line), and for I_0_ = 4 μA/cm^2^ and membrane patches of 300 μm^2^ (dashed purple line) and 600 μm^2^ (dashed orange line); the signal is a sinusoid with amplitude V_ES_ = 500 μV and frequency f = 150 Hz; the exogenous noise is a zero mean Gaussian process with a Lorentzian spectrum (ω_c_ = 2.5×10^3^ rad/s)**.

Another effect of the exogenous noise is a considerable increase of the number of spikes per second and, consequently, of the power associated with the spike sequence (U(t)). Unlike the SNR, these quantities exhibit a monotonic increase with D, approaching asymptotic values, as shown in Figure 8 for the number of spikes per second.

This means that the increase in firing activity does not necessary imply an improved encoding capability. Indeed, although a minimum number of spikes per second is required to efficiently encode a 150 Hz sinusoidal signal, if noise exceeds the optimum level, neuron activity is dominated by noise and less correlated with the signal, in agreement with the SR phenomenon (Gammaitoni, 1998; Moss et al., 2004; McDonnell and Abbott, 2009).

Table 3 shows the number of spikes per second and the power assumed by U(t) corresponding to D_opt_, compared to their values in the absence of the exogenous noise. In all cases the number of spikes at the optimum noise level is between 28 and 38, with a power from 1.4 to 1.8 mV^2^, suggesting that these numbers of spikes per second can efficiently encode a sinusoid at 150 Hz in a subthreshold neuron. Thus, it is not surprising that the model with a 600 μm^2^ area and I_0_ = 4 μA/cm^2^ shows good encoding properties at 150 Hz (SNR = 3) (see Figure 6C) with 30 spikes per second (see Figure 4).

**Table 3:** **Number of spikes per second and power of U(t) for the neuron model without and with the exogenous noise at the optimum level D_opt_**.

**Patch area (μm^2^)**	**Input current (μA/cm^2^)**	**Spikes/s**		**Power (mV^2^)**	
		**Without exogenous noise**	**With optimum exogenous noise**	**Without exogenous noise**	**With optimum exogenous noise**
200	2	14.6±0.3	31.9±0.2	0.68	1.73
300	2	7.1±0.9	37.8±0.2	0.32	1.54
300	4	22.3±0.4	28.6±0.2	1.06	1.38
600	4	9.4±0.9	35.7±0.2	0.47	1.84

## Discussion and conclusions

In this work, moving from the original HH description, a stochastic neuron model has been developed and the presence of an exogenous signal and noise, representative of a possible electric or magnetic stimulation, has been added. The firing activity of the model has been studied for three different levels of channel noise, corresponding to different patch areas (200, 300, and 600 μm^2^) and for three input currents, representative of a “healthy” neuron (I_0_ = 7 μA/cm^2^), an “impaired” (I_0_ = 4 μA/cm^2^), neuron and a “severely impaired” neuron (I_0_ = 2 μA/cm^2^), where the afferent stimulation is reduced due to aging or degenerative diseases.

Results indicate that the “impaired” neurons are much less excited (less than 10 spikes/s for a patch of 600 μm^2^, I_0_ equal to 2 and 4 μA/cm^2^, and a patch of 300 μm^2^, I_0_ = 2 μA/cm^2^), suggesting reduced performances in signal encoding and processing.

The presence of the exogenous sinusoidal signal (V_ES_ = 500 μV; f = [10–500] Hz) does not significantly change the firing frequency (Figure 4), confirming that the neuron does not use the frequency encoding paradigm to sense such low-level alternate signals. On the contrary, the PSD of the spiking sequence U(t) reveals the presence of a peak corresponding to the signal frequency, suggesting a time encoding mechanism (Figure 3).

Results of the SNR, chosen as a measure of signal encoding, show a strong sensitivity to the signal frequency, as suggested by the bell-shaped curves of Figures 5, 6. For example, the “healthy” neuron does not sense signals at 10 or 500 Hz (SNR = 1), whereas, for a frequency of 150 Hz, the SNR can be as high as 6.8 (Figure 5). Such frequency sensitivity significantly depends on the stimulation current I_0_. This result confirms that the encoding capability of the neuron can be strongly altered by a decrease in the presynaptic input current.

The neuron size, and thus the endogenous noise, mainly affects the maximum SNR, indicating that the encoding capability is a function of the type of neuron (e.g., auditory fiber, bushy cell, cortical neuron). Interestingly, the endogenous noise reduces the encoding capability of the “healthy” neuron but facilitates the signal detection in “impaired neurons,” suggesting a similar behavior if the noise is delivered externally together with the stimulating sinusoidal signal.

An exogenous voltage noise, modeled as a zero-mean Gaussian process with a Lorentzian spectrum and variable power (D), has been added to the models of the “impaired” neurons. The SNR obtained as a function of the noise power exhibits a typical bell shaped behavior with a maximum value corresponding to a well-defined value of D (D_opt_), which depends on the values considered for the I_0_ and patch area.

The exogenous noise at the optimum levels can significantly increase the SNR of the “impaired” neurons at 150 Hz (up to 58%, depending on the neuron size and the impairment level). Since the neuron model takes advantage of the noise to improve the detection of a weak sinusoidal input signal, the observed behavior can be attributed to the well-known SR phenomenon (Gammaitoni, 1998; Moss et al., 2004; McDonnell and Abbott, 2009) (Figure 7).

Beside the improvement in the SNR the exogenous noise induces an increased firing activity that for D_opt_ is characterized by 28–38 spikes/s.

These results are significant if considered as a proof of concept on how to use artificial exogenous noise to restore the functionalities of signal detection and processing in impaired neuronal systems.

This is a first step toward the optimization of specific biomedical applications such as cochlear prosthesis (Morse and Roper, 2000; Rattay, 2000; Stocks et al., 2002; Rubinstein and Hong, 2003) and ICMS (Overstreet et al., 2013).

Further developments of this work could be the optimization of the exogenous noise, in terms of the spectrum shape and/or the kind of stochastic process and a more accurate description of different neurons in terms of type and number of channels.

Finally, depending on the particular biomedical application, the typical waveform and amplitude of the exogenous voltage signal superimposed on the physiological transmembrane potential can be calculated using dosimetric (Liberti et al., 2007; Maggio et al., 2009, 2010; Paffi et al., 2013a,c, 2015) and microdosimetric techniques (Liberti et al., 2009b; Merla et al., 2010, 2011, 2012; Denzi et al., 2013, 2015), as described in the integrated methodology proposed by Apollonio et al. (2000, 2013).

## Author contributions

ML and FA developed the underlying concept of this study, with contributions from AP. AP and FC prepared computer-simulation codes and methods, and AP, FC, and ML carried out the analysis. All authors discussed results, interpreted data, and formulated findings. AP wrote the manuscript, with some contributions from FA and ML.

### Conflict of interest statement

The authors declare that the research was conducted in the absence of any commercial or financial relationships that could be construed as a potential conflict of interest.
